# The Impact of Language Diversity on Knowledge Sharing Within International University Research Teams: Evidence From TED Project

**DOI:** 10.3389/fpsyg.2022.879154

**Published:** 2022-04-21

**Authors:** Rossella Canestrino, Pierpaolo Magliocca, Yang Li

**Affiliations:** ^1^Department of Management and Quantitative Studies, University of Naples Parthenope, Naples, Italy; ^2^Department of Humanities, Literature, Cultural Heritage, and Education Sciences, University of Foggia, Foggia, Italy; ^3^School of Cultural Creativity and Management, Communication University of Zhejiang, Hangzhou, China

**Keywords:** language diversity, knowledge sharing, university research team, TED project, cultural diversity

## Abstract

In today’s knowledge economy, knowledge and knowledge sharing are fundamental for organizations to achieve competitiveness and for individuals to strengthen their innovation capabilities. Knowledge sharing is a complex language-based activity; language affects how individuals communicate and relate. The growth in international collaborations and the increasing number of diverse teams affect knowledge sharing because individuals engage in daily knowledge activities in a language they are not native speakers. Understanding the challenges they face, and how they manage the emerging difficulties is the main aim of this manuscript. For this purpose, an explorative case study was conducted in an international university research project, namely the TED project. Both interviews and direct observations were employed to understand the phenomenon better and deliberately triangulate data and improve validity. Results show that non-native language use determines the emergence of different language proficiency, depending on the nature of the knowledge domain–job-related vs. non-job-related. Within non-job-related knowledge domains, the lack of linguistic abilities, summed to the perceived cultural diversities, mainly affects people’s propensity to engage in personal and more intense social relationships. Under such circumstances, tacit knowledge sharing is reduced with negative consequences on the project’s long-term innovative performance. Since the project is still running, detecting language challenges will allow the partners to design and apply effective measures to support cooperation with language and cultural barriers. Among them, *code switching*, adopted by “bridge” actors, already emerges as tool supporting communication and knowledge exchange.

## Introduction

Due to the globalization of both research and business activities, the most recent changes in the organizational workforce aroused the interest of researchers and practitioners in language diversity and its impact on knowledge sharing in multinational and multilingual contexts (Alinasab et al., 2021; Debellis et al., 2021). Remarkably, Ahmad (2018, 2017) considers knowledge sharing a language-based activity and the use of non-native language an ambiguous and costly process, eroding some of the advantages of the process. Similarly, Tenzer et al. (2021) sustain that language diversity negatively affects communication in multinational teams, thus influencing knowledge processing. Hence, language diversity is an important and influential factor in knowledge sharing in international contexts, teamwork, and organizations. However, the relationship between language diversity and knowledge sharing has not been explored in detail; the available literature mainly focuses on business and entrepreneurial environments.

Knowledge about knowledge sharing within international university research teams is still lacking (incomplete) and fails to incorporate relevant insights and perspectives (inadequacy) when dealing with language diversity. To date, most of the available studies deal with entrepreneurial and business multinational teams (Henderson, 2005; Ahmad, 2018, 2017; Tenzer et al., 2021), suggesting different adjustment approaches to remove the obstacles caused by the use of non-native language.

In order to fill this gap, this manuscript aims to explore how language diversity—as a measurable component of culture—affects *knowledge sharing* and *knowledge sharing behavior* within international research teams.

From a social psychological perspective, knowledge sharing is theorized as a process of social interaction, based on the knowledge exchange among individuals consciously acting to share, with others, what they already know (Davenport and Prusak, 1998; Caputo, 2017; Del Giudice et al., 2017).

Scholars are used to investigating how individual and organizational factors support, or mine, knowledge sharing within organizations or teams (Bock and Kim, 2002; Ipe, 2003; Canestrino and Magliocca, 2016; Scuotto et al., 2020). Notably, based on a systematic literature review, Intezari et al. (2017) propose an integrative framework for studying and practicing organizational knowledge culture, detailing the factors affecting, among the others, people’s willingness to explore and share knowledge (social interactions, openness in communication, trust, and perception of knowledge). Moreover, Welch and Welch (2008) focus on international knowledge transfer, examining the range of influences—cost, transfer medium, teams, networks, trust, staff movements, and motivation—that language has on the process.

Language diversity is well-known in the literature for its potential to disrupt social interaction (Mcall, 2003). Language differences have been found to cause dysfunctional group formations, social fragmentation, and lower individuals’ rhetorical capacities in diverse settings (Feely and Harzing, 2003). Detailing its role in sharing knowledge among the members of international university research teams is the main aim of our work. Accordingly, this paper investigates the impact of language diversity on communication and knowledge sharing in the multicultural university team belonging to the so-called TED project (Teaching Digital Entrepreneurship). TED is a project financed in 2020 under the Erasmus+ European Project, involving seven university partners from five different cultural and linguistic areas (Austria, Italy, Poland, Spain, and Ukraine), aiming to reinforce collaboration and knowledge sharing among the parties. The project’s final goals are updating knowledge in digitalization and advancing a shared, international curriculum about digital entrepreneurship. Thus, overcoming the barriers arising from language diversity is essential to establish effective communication among the partners and foster knowledge sharing.

In line with the mentioned, this paper aims to detect which challenges the TED partners face in terms of knowledge sharing and how they manage the emerging language difficulties.

In order to get the final goal of our investigation, we will employ a semi-structured qualitative research strategy, which provides consistency in crucial questions and enables robust theory building through constant comparison among informants. Data generation will follow the protocol detailed by Tenzer et al. (2021). Data analysis and interpretation will base on the approach Gioia et al. (2013) recommended to prevent the loss of information as possible.

Since contemporary science characterizes the flourishing of international research teams, our study will provide valuable contributions to the psychology and management fields. Since collaborations across organizational, disciplinary, and cultural boundaries extend the chances of sharing and creating new and valuable knowledge (Dusdal et al., 2021), inspecting how language diversity affects team dynamics is worth mentioning.

The manuscript is organized as follow: the first section provides an overview of the scientific literature dealing with Knowledge Sharing (KS) within university research teams and the role played by the language diversity. Then, section two details the adopted methodology with reference to both materials and research procedure. Findings are illustrated and discussed in sections three and four. Finally, conclusions and limitations of the manuscript are reported in the last section.

## Literature Review

### Knowledge Sharing Within International University Research Teams

Both globalization and the fierce economic competition impel firms to innovate continuously to maintain competitiveness. Depending on the above, knowledge has become one of the most strategically significant resources. There is an increasing recognition that creating, transferring, and sharing knowledge is crucial to get firms’ competitive advantage (Grant, 1996; Spender and Grant, 1996; Davenport and Prusak, 1998; Foss and Pedersen, 2002). Researchers do not reach a consensus on the distinctions between knowledge and information. Notably, Machlup (1980), Kogut and Zander (1992), and Zander and Kogut (1995) consider knowledge much more than a piece of information, knowledge including information and know-how. By contrast, Huber (1991); Makhija and Ganesh (1997), and Bartol and Srivastava (2002) do not find any practical advantage in distinguishing knowledge and information when dealing with knowledge-sharing research. Under this consideration, we use the terms knowledge and information interchangeably since this research focuses on KS within international university research teams.

Knowledge sharing refers to making knowledge available to others who participate in this process of exchange. Participants do not receive any compulsory pressures; thus, KS emerges as a conscious and voluntary act (Davenport, 1997) developing in collaborative settings (Hendriks, 1999). Different from the knowledge transfer, KS includes providing knowledge to others (transfer of knowledge) and searching for knowledge from others, thus always resulting in an exchange mechanism (Wang and Noe, 2010).

Scholars document the benefits of KS at both individual and organizational levels. Sharing knowledge among people contributes to individual learning (Andrews and Delahaye, 2000; Nidumolu et al., 2001), supporting creativity and the diffusion of innovative ideas within the organization (Armbrecht et al., 2001; Caputo et al., 2021). Employees engaging in KS activities are more likely to find solutions to their complex problems (Ghaznavi et al., 2011); cooperation and discussions among colleagues enhance employees’ productive capacity, ultimately affecting organizational performance (Ahmad, 2017). Moreover, collaboration among employees provides the linkage between individual and organizational learning, mainly because individual learning contributes to organizational learning, supporting the firm’s competitive advantage (Hendriks, 1999). Despite this, KS is a complex process influenced by several obstacles (Khelladi et al., 2022). It requires effective knowledge management strategies aiming to neutralize or limit the “*problems that lie in the intersection of organization and knowledge processes*” (Foss, 2007, p. 39).

Knowledge sharing is even more crucial in university research teams (Hernaus et al., 2019) since the universities are expected to support the improvement of knowledge in society. Notably, many changes have affected universities for the last years, universities experiencing the transition from the traditional mission of education and research to a broad assignment involving the contribution to the economic development by means of the knowledge transfer of its research results (Feola et al., 2020). In line with the mentioned, scholars recognize entrepreneurial universities (Etzkowitz et al., 2000; Mian, 2011; Fayolle and Redford, 2014) as key actors of economic growth and wealth creation, embedded in various nested and interdependent competitions (Krücken, 2021). Consequently, formal evaluations, performance measures, and comparative indicators are introduced to assess academics’ capacity to get research results. Therefore, academics are forced to compete to achieve promotions, publications, and funds for projects (Ballesteros-Rodríguez et al., 2020; Dusdal and Powell, 2021). Under such circumstances, the collaboration between and among researchers is increasingly emphasized at both the national and international levels. Thus, “*contemporary science is marked by expanding, and diverse forms of teamwork*” (Dusdal and Powell, 2021, p. 235), and university research teams establish to succeed in this new learning race (Powell, 2020). Notably, university research teams are communities of researchers working together to get a defined knowledge aim (Katz and Martin, 1997). In so doing, team members develop research activities, share materials, and financial resources, exchanging ideas and expertise. In line with the mentioned, university research teams could be considered the ideal background for KS since all members can easily access the knowledge of others and expand their cognitive abilities (Oliveira et al., 2019). Despite this, many features can mine the effectiveness of KS within university research teams, eroding the benefits of the process. Knowledge exchange in university research teams is not an automatic route but a complex process of interaction among different individuals, threatened by multiple factors.

**FIGURE 1:**
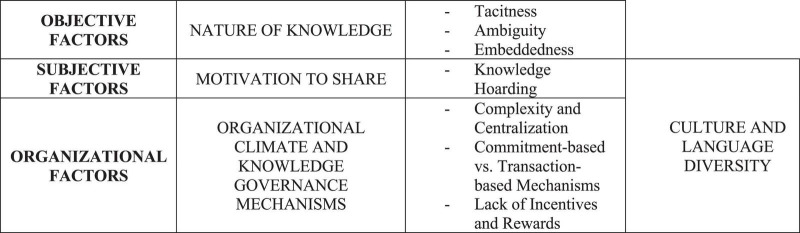
The Obstacles to KS within Organizations. Source: Authors’ elaboration.

Based on a review of the theories and research related to knowledge sharing, Ipe (2003) identified four major factors that influence knowledge sharing between individuals in the organizations: the nature of knowledge, motivation to share, opportunities to share, and the culture of the work environment. Organizational climate and knowledge governance mechanisms—complexity and centralization; commitment-based vs. transaction-based mechanisms; incentives and rewards–also add to the obstacles to KS (Wang and Noe, 2010; Canestrino and Magliocca, 2019, 2016) (Figure 1).

The nature of knowledge—tacit vs. explicit; simple vs. complex; and independent vs. systemic (Kogut and Zander, 1992; Nonaka and Takeuchi, 1995)–and its “casual ambiguity” represent the main objective obstacles to the KS process (Canestrino and Magliocca, 2016). Starting from Polanyi’s conceptualization of tacit knowledge (Polanyi, 1962), Nonaka and Takeuchi (1995) widened the notion, opposing to the tacit knowledge its natural reverse, namely the explicit knowledge. Nowadays, the dominant classification of knowledge within the organizations divides it into two types, tacit and explicit. Explicit knowledge can be easily codified and transferred by documents or other standardized tools. Tacit knowledge, on the contrary, cannot be easily transferred because of its embeddedness in the human mind and into the organizational routines (Simonin, 1999). Unlike explicit knowledge found in manuals or books, tacit knowledge is not easy to articulate, codify, or transfer directly from one person to another. Consequently, sharing tacit knowledge requires close interactions between and among the organization’s members, storytelling, traditions, and routines (Von Krogh et al., 2000). Nevertheless, compared to the explicit, tacit knowledge is more valuable for gaining firms’ competitive advantage since it is difficult to imitate and replicate (Grant, 1996).

Motivation to share belongs to the so-called subjective obstacles to KS (Canestrino and Magliocca, 2016). Notably, people without a solid personal motivation are not likely to share knowledge (Stenmark, 2001); thus, a Knowledge Hoarding (KH) establishes, threatening the continuity of the organization’s knowledge base and innovativeness (Anaza and Nowlin, 2017; Trusson et al., 2017; Bilginoğlu, 2019; Canestrino and Magliocca, 2019; Khelladi et al., 2022). KH refers to “*an individual’s deliberate and strategic concealment of knowledge and information or the fact that they may possess relevant knowledge or information*” (Evans et al., 2015, p. 495). In their “knowledge hostility model,” Husted and Michailova (2002) examined the reason why knowledge senders may choose to hoard their knowledge. According to the authors, individuals wish to protect what they know since their competitive advantage relates to “*the quality and value of the knowledge he or she possesses’*’ (Husted and Michailova, 2002, p. 65). Since knowledge is often considered “hard-won,” people may develop a strong feeling of personal ownership, thus collecting and storing information that could be useful in the future (Husted et al., 2012; Anaza and Nowlin, 2017). Reasons for hoarding were also examined by Du Plessis (2005), Willem and Buelens (2007), Yang (2007), Kuo and Young (2008), Yamao et al. (2009), Tseng (2010), and Wang and Noe (2010). They all recognize that people hoard knowledge because they wish to retain power and/or control. Similarly, the competitive behaviors of academics can reduce their willingness to share knowledge with colleagues to get superior research performance (Hernaus et al., 2019) even though the distribution of power matters in organizations. Ghoshal and Bartlett (1994) argued that trust facilitates learning and decisions to exchange knowledge among individuals. Mainly, trust alleviates the negative effect of perceived costs on sharing (Kankanhalli et al., 2005), diffusing the idea that others are contributing equally to the community knowledge growth and that no one is opportunistically exploiting the partners’ cooperative efforts (Canestrino and Magliocca, 2019). In this context, the role of the team leader is crucial in creating a trustful environment that encourages team members to share their ideas and knowledge (Liu and Phillips, 2011; Castellano et al., 2017, 2021).

Organizational climate, knowledge governance mechanisms, and the lack of incentives and rewards, also influence individuals’ and employees’ knowledge-sharing behavior. Notably, an organizational climate that emphasizes individual competition may limit KS. In contrast, cooperation supports trust-building among the parties, intensifies the frequency and the intensity of the relationship, shaping the conditions for knowledge sharing within the firms and the teams, as well (Wang, 2004; Willem and Scarbrough, 2006; Schepers and Van den Berg, 2007; Wang and Noe, 2010). In Abili et al. (2011) analyzed the impact of complexity and centralization on the flow of information among organizations’ employees. According to their findings, the less centralized structure is, the more KS occurs, suggesting managers create open workspaces (Huang et al., 2013) and encourage more informal meetings (Wang and Noe, 2010) to increase communication flow throughout the organization (Ali et al., 2018). Notably, both formal (training programs, structured work teams, and technology-based systems) and informal (personal relationships and social networks) facilitate learning and the exchange of knowledge among people (Brown and Duguid, 1991; Nahapiet and Ghoshal, 1998; Ipe, 2003; Khelladi et al., 2022). Focusing on how knowledge governance mechanisms affect the KS within organizations, Husted et al. (2012) found that “*the use of transaction-based mechanisms promotes knowledge-sharing hostility by strengthening individuals’ reasons for hoarding and rejecting external knowledge, and negatively affects individuals’ attitudes toward sharing knowledge about mistakes*” (p. 768). By contrast, employing commitment-based mechanisms diminishes knowledge-sharing hostility among individuals, thus supporting KS processes. Finally, both concrete and perceived rewards and penalties for individuals who share and do not share knowledge influence the KS process (Ipe, 2003). Yao et al. (2007) suggest that the lack of incentives is the most important barrier to knowledge sharing across cultures. Many scholars (Hansen et al., 1999; Liebowitz, 2004; Nelson et al., 2006) also recommend using rewards and other incentives to sustain KS and develop a supportive knowledge culture. Despite the mentioned, no consensus arises from the results of empirical researchers aiming to investigate the positive linkage between incentives (as extrinsic motivation) and KS (Wang and Noe, 2010). Thus, as Ali et al. (2018) recently demonstrated, the link between rewards and KS varies across the business environment.

Most of the examined obstacles to KS affect the knowledge exchange within university research teams. As mentioned, KS among university team members is not automatic, mainly because of the competitive behaviors of academics and the expansion of the team scale, the last resulting in the reduction of frequent communications among the parties (Xia and Ya, 2012) and a growth of cultural and linguistic diversity (Henderson, 2005; Ahmad, 2017; Dusdal et al., 2021). KS requires researchers to become involved in joint discussions and exchanging ideas (Han et al., 2014). Interaction and communications among the team members are necessary to promote the commitment, trust, and cohesion that enable knowledge exchange (Zboralski, 2009). If communications reduce, mutual understanding is more challenging to establish, negatively impacting the team learning process and performance (Xia and Ya, 2012).

Moreover, regardless of what the university team does to reach its research aims, culture and language diversities affect how knowledge is communicated, diffused, and shared among the members. Schein (1985) defined culture as a “*pattern of basic assumptions*” (p. 9) that is developed by a group that faces everyday problems. Moreover, culture also defines “*how members of a group take action, how they determine what is relevant information, and when they have enough of it, to determine whether to act and what to do*” (Schein, 1985, p. 89). The linkage between culture and knowledge is evident in the above definition and stressed by many authors in knowledge management studies (Canestrino and Magliocca, 2019; Canestrino et al., 2020; Orlando et al., 2020; Cillo et al., 2021). Notably, De Long and Fahey (2000) identified how organizational culture influences KS, namely by (a) shaping the assumptions about what knowledge is, (b) defining the relationship between individual and organizational knowledge, (c) creating the context for social interaction; and finally, by (d) defining which type of knowledge will be used in a particular situation.

Similarly, language as a vehicle of knowledge is widely accepted in the literature. Language affects knowledge creation and sharing and provides the base for social interaction (Choo, 1998; Renzl, 2007). Because of the expansion of collaboration across organizational and cultural, university research teams are expected to manage the challenges arising from cultural and linguistic diversity. Operating across different cultures and languages is a crucial feature of contemporary science since it extends the chances of discovery. However, compared to cultural diversity in international teams, which was widely discussed among the scholars (Iles and Hayers, 1997; Smith and Berg, 1997; Maznevski and DiStefano, 2000), literature devoted relatively little attention to language diversity, which is the focus of our investigation.

### The Impact of Language Diversity

The linkage between language and KS has attracted the attention of scholars in recent years (Welch and Welch, 2008; Schomaker and Zaheer, 2014; Ahmad and Widén, 2015). Language is expected to significantly influence any organization characterized by linguistic variations (Lauring and Selmer, 2011). As Chomsky (1992) noted, language affects many aspects of human life; language provides the context within which people learn and know. Notably, language plays a crucial role in constructing knowledge, serving as a vehicle of both thoughts and meanings (Renzl, 2007). In line with the mentioned, the rise of multilingual organizations and research teams fostered the debate about the effectiveness of knowledge production and sharing within teams composed of different languages. “*The presence of a multitude of speakers of different native languages*” (Lauring and Selmer, 2012, p. 157) is defined as language diversity. As Henderson (2005) noted, language diversity may be responsible for communications breakdowns caused by weak language proficiency. Lack of linguistic skills in a specific language (mainly the official language adopted within a given group or within an organization) may lead to misunderstandings and incapacity to share knowledge (Makela et al., 2007; Welch and Welch, 2008).

Moreover, language diversity is reported to challenge the development of social relationships, language weaknesses being responsible for a linguistic sidelining, with people choosing to reduce their involvement in team communication (Tenzer et al., 2021). Within knowledge management, Henderson (2005) analyzed language diversity from a socio-linguistic perspective, demonstrating it has a crucial role in socialization processes and team building, affecting both communications among the parties and mutual perception. The author noted that language diversity in international management teams poses many challenges for both native and non-native speakers. Depending on it, managers and leaders should detect language diversity to address its main consequences. Within the field of knowledge management, Ahmad and Widén (2018) examined the process of KS in non-native language contexts, detailing the strategies adopted by the employees to deal with problems of KS. One of the significant findings of their study is that employees feel that knowledge sharing in a non-native language setting is a costly activity. Lack of linguistic proficiency or knowledge deficiency (lack of expertise) makes it difficult to manage communications, impelling people to adopt some strategies—discourse adjustments, media adjustments, and language adjustments—to deal with linguistic differences.

Similarly, the two language practices known as *code-switching* and *convergence* were examined to understand their impact on individual KS in multilingual organizations (Ahmad and Widén, 2018). More recently, Tenzer et al. (2021) investigated how language diversity affects communication and knowledge processing in multinational teams. Based on empirical research, the authors show that two kinds of language barriers—evident and hidden barriers—negatively affect participation and sense-making in multinational teams. These barriers mine the effectiveness of more complex knowledge processing activities, asking for deepening explorations.

Despite scholars recognizing the relevance of language diversity in KS, research on the topic is still at its initial stage, and empirical evidence mainly refers to multinational organizations, where the perceptions of leaders, managers, and employees are usually mixed with synthesizing the results. In this regard, focusing on the impact of language diversity on KS within an international research team represents our current effort of enriching the literature about the topic.

## Materials and Methods

### Study’s Context

Teaching Digital Entrepreneurship project in the context of this investigation about the impact of language diversity on knowledge sharing in the university research team. Notably, TED is a project financed in 2020 under the Erasmus+ European Project, involving seven university partners mainly from UE (with the only exception of Ukraine). The Krakow University of Economics (CUE) (Poland) leads the project. University of Jaèn (UJ) (Spain); the Wirtschaftsuniversitat Wien (WU) (Austria); the Boris Grinchenko Kyiv (BGU) University (Ukraine) and three Italian Universities—Parthenope University of Naples (UP), University of Salerno (US), and University of Foggia (UF)—belong to the international networks. The project started on September 1, 2020, and it is still running. The duration time of the project is 36 months; thus, it will end on August 31, 2023. The project aims to fill the gap between the competencies required (to both the individuals and the organizations) to compete in a digitalized world and the High Educational teaching programmes, mainly addressed to transfer knowledge to start and manage a traditional business. Accordingly, updating knowledge in digitalization and advancing a shared, international curriculum about digital entrepreneurship are the main expected results of the project. The curriculum will be equipped with teaching guidelines, a textbook, and a casebook, allowing for new technology inclusion into education programs. These materials result from the collaboration among all the involved partners, called to share ideas and knowledge to build a new teaching methodology in a very advanced research field.

**FIGURE 2:**
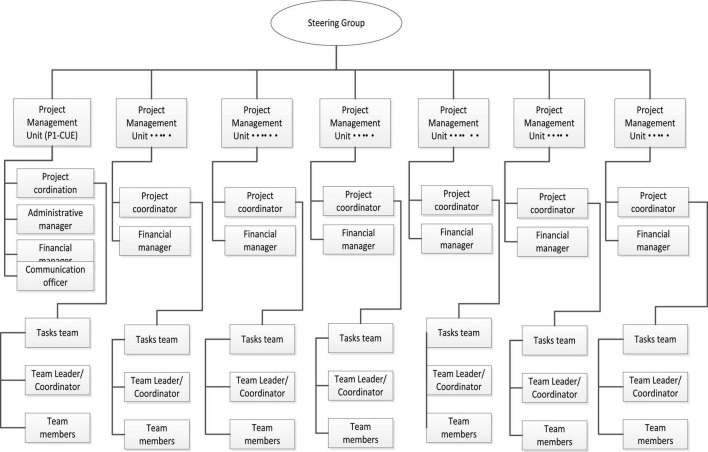
TED project structure.

Shows the structure for the whole project. Each partner joined the project with 2–4 members directly involved in the different tasks. For this research, only the partners (project coordinator, tasks’ leaders/coordinators, and team members) directly involved in the research activities were selected for investigation (Figure 2).

Under the project schedule, official meetings (kick-off meetings, follow-up meetings, mid-term meetings, and retrospective meetings) should be organized to plan and check the team research activities. Because of Pandemic, the kick-off meeting was organized online in September 2020. The follow-up meeting, hosted in Cracow on September 2021, followed it. At the time of this investigation, one project output (O2 = teaching guidelines for digital entrepreneurship) was completed. O1 (Textbook “Doing Business Digitally”) and O3 (Casebook “How to do business in the digital era?”) were completed in their final draft stage.

Team members met online every month to discuss the research issues and project advancements. English is used as a common language for official communication and formal and informal meetings. Since partners come from five different countries, speaking a different language—Austria, Italy, Poland, Spain, and Ukraine—the project is suitable for the research purpose advanced in this manuscript. Detecting the challenges language diversity determines in knowledge sharing within university research teams is explorative. Thus, adopting a single case study method is deemed a suitable research strategy in such circumstances (Eisenhardt, 1989; Yin, 2009).

### Data Collection and Procedure

In total, 13 in-depth interviews were conducted with the representatives of the five different languages participating in the project as research members/leaders/coordinators. Three further interviews were conducted as a confirmatory step. Since information begins to repeat and no additional issues were identified, data collection becomes redundant (Strauss and Corbin, 1994; Guest et al., 2006; Kerr et al., 2010). Thus, in this analysis, data saturation was reached with 13 interviews. It is in line with the Italian universities’ main representativeness, which accounts for the majority of the participants in the project. The inclusion of interviewees from all the different languages enhanced the subject diversity and unit triangulation of the data (Marschan-Piekkari et al., 1999). Additional information was collected through direct observations (two of the authors of this manuscript belong to the research project, and they actively participated in all the virtual and physical meetings organized by the international team), in line with Makela et al. (2007). Furthermore, project documentation, research notes, intranet and internet data, and archival data were used for triangulation. Provides detailed information on the research design (Table 1).

**TABLE 1:** Research design.

In-depth interviews				Supplementary data collection

Participants and their native language				
	
Country	Native language	N. Interviews	Team members	
Austria	German	2	2	Direct observation; Project documentation; Research notes; Intranet and internet data; Archival data
Italy	Italian	5	13*	
Poland	Polish	2	3	
Spain	Spanish	1	2	
Ukraine	Ukrainian	3	3	
Total		13	23	

*Here, the total number refers to the number of researchers belonging to the three Italian Universities—Parthenope University, University of Foggia, and the University of Salerno. Representatives from the three Universities were interviewed until the saturation of the collected information.

None of the interviewed are English native speakers. An invitation to participate in the survey was e-mailed to the TED members at the beginning of December 2021. Disposals were scheduled following each member’s availability; thus, interviews were managed from the middle of December 2021 and January 2022. All of the 14 interviews were conducted through the university web platform and were video-recorded. The average interview time was 33 min, the shortest was 21 min, and the longest was 50 min. As Yin (2009) recommended, a research protocol—including the interview guide and the procedure guide—was planned before engaging in the research. The interview guide, based on open-ended questions–was developed following Ahmad (2018). Questions mainly focus on the experience developed within the project, how language diversity (use of non-native language) affected social interaction and KS, and how this is perceived to mine the team’s ability to achieve the project aims. Notably, participants were asked to provide practical examples of the difficulties they faced during online and face-to-face meetings to contextualize both the relational and the learning dynamics characterizing the project (Augier et al., 2018). Questions about the use of non-native language during online and physical meetings were added to clarify how the use of technology supports or mines social interactions and KS. It means asking the interviewees to compare the same activities (in a non-native language) in a physical and virtual environment. The complete interview guide is reported in Supplementary Appendix 1.

The intertwining of interviews, direct observation, and data collection allowed the interviewer to further elaborate on the emerging themes and fine-tune the questions’ orientation. Interviews were transcribed verbatim, and NVivo 11 was used for qualitative data analysis. Data analysis and interpretation followed the approach recommended by Gioia et al. (2013). This approach helped prevent the loss of information by coding the data corpus (informants’ voices) as first-order codes before aggregating them to second-order themes (abstract concepts from the first-order categories) and finally identifying the aggregate dimensions (theoretical themes). Coding was undertaken conservatively, being based only on what the data explicated. By comparing the codes, similarities and differences were identified, and the number of codes was reduced. In particular, each researcher separately coded the concepts of the first order, carried out consistency checks, and carefully coded all the textual data, thus allowing multiple coding of each textual unit, thereby guaranteeing data triangulation.

## Findings

The section is structured by the thematic blocks that emerged from our investigations, mainly referring to the role of *language diversity in communication and KS within the international university research team*, the *language variations in KS*, and the *role of technology (ex.: virtual communications).* The presentation of findings and their advance in a broad theoretical model reflect the process of inductive mid-range theory building.

### The Role of Language Diversity in Communication and Knowledge Sharing Within Teaching Digital Entrepreneurship

The large majority of the interviewed agree they have not experienced strong KS difficulties directly connected to the use of English in pursuing the project’s goals, nor in the related job communication. This result appears obvious, as all the partners have been embedded in research activities and international-related tasks for many years (from three to more than 20 years). Additionally, all of them use English every day to manage jobs–mainly to read papers; send and receive e-mails. English language spoken activities are less frequent, varying from every day to every three months. As some interviewed reported:

“*Inside our project, I have no strong language problems, since all the participants are very qualified in English…”*

*“I think we are quite advanced…in my opinion, we do have not many difficulties because we can always ask each other: ok, what do you mean? I do not understand. Can you please explain me?”*

Despite this, individuals from various linguistic backgrounds require members to spend more time listening to the partners before understanding them. Notably, different accents are perceived as the main obstacle to communication within the team, requiring a time-spending relationship to overcome it.

As some interviewed reported:

*“… since different countries are involved, and partners have different pronunciations, I usually need to listen twice or three times to one person who is speaking with a specific accent, and after that, it’s easier for me to understand him.”*

*“Accent is totally different from one person to another; sometimes I’m challenged by the accent of person to whom I’m speaking.”*

Moreover, some difficulties in understanding and KS are reported depending more on “cultural matters” than on the partners’ language abilities.

*“I got troubles in understanding what some partners are explaining in terms of “systems,” “education,” “institutions,” etc. … but it does not depend on the English, but on the meaning, they give to such kind of words… I’m not able to much these concepts with the context I live in; thus, it is more difficult for me to understand them….”*

*“Some partners are very “formal” when communicating… It’s not a language problem…it depends on the individuals’ behavior and on how they relate to all the others…I’m a very informal person… I do not feel comfortable with such formal behavior.”*

*“I perceive some weaknesses in the project related to how some activities were managed…I mean in terms of deadlines, scheduling, and coordination. There was not a problem of English proficiency, but a cultural one… Leadership was different when jumping from one task to another, affecting how we work on the outcomes.”*

Language embodies the specific culture and strongly interacts with other cultural components—such as values, cognitive schema, and demeanor. Despite this, our empirical evidence shows that language has a distinct influence on the functioning of the international research team and the KS among the partners. Our results find support in the studies by Hambrick et al. (1998) and Welch and Welch (2008), according to which language is deserving of investigation in its own right. However, suppose we accept that “*Language has an importance above and beyond the “embeddedness-in-culture” perspective”* (Welch and Welch, 2008, p. 341). In that case, we should agree on the existence of a double challenge to KS within the examined international research teams. The first challenge depends on the lack of language proficiency, the last one responsible for the more visible difficulties in communication and socialization. The second challenge is less visible and depends on the different meanings/interpretations that people attribute to certain words or sentences. Of course, people use language to construct reality and versions of the social world (Renzl, 2007), language acting as a symbolic representation of society. This symbolic representation arises from history, traditions, heritage, functioning of national systems, and how society is structured; thus, it may vary from country to country, affecting how non-native language speakers interpret some notions.

Take, as an example, the concept of “System” and how Roberto Saviano defined it in one of its most famous books: *Gomorra.* Saviano (2006) is a hybrid text between journalism and fiction that describes Naples’s Camorra and its international ramifications. The text is widely populated by culture-bound concepts and implicit meanings, which further complicates the translation process, and one of them is the concept of “*System*.” In the south-Italian culture, the “System” stands for an institutionalized and hierarchical crime organization, alternatively used to identify the Camorra (that is different from the Italian mafia) (Cavaliere, 2010). In his book, Saviano devotes entire pages to explaining how the *camorristi* mark their membership to the “*System*.” Without this detailed description, non-native speakers would be unable to perceive its meaning simply because the Camorra and the “*System*” are contextual phenomena, far from the English-speaking target reader, and do not exist elsewhere.

One of the TED research team’s first challenges was clarifying the notion of “digital entrepreneurship” in a familiar and shared way, since what “digital entrepreneurship” is and how “digital entrepreneurship” develops may change according to national rules and contextual features. Interaction and debate are crucial for constructing a familiar shared meaning about the topic.

By contrast, language proficiency, limitations in vocabulary, and lexical shortcomings are perceived to affect personal and informal communication and social interactions. The last ones are limited as an extreme consequence of low English fluency. Thus, in some circumstances, some team members’ sidelining behavior was observed, limiting their speaking up or joining the social team activities.

*“Sometimes it’s hard to say something like jokes… I’m a very ironic person, and it’s very difficult to translate what you mean because you are not so fluent in English… but it’s about informal communication. It does not refer to substantive matters of the project.”*

“*When we discuss about the cousin and the food, I have not enough knowledge about all the names of vegetables, etc. … For me, speaking English is easier when referring to job activities and topics related to my job.”*

“*I’m aware of my English limits, and I feel much more comfortable speaking about project-related issues since I’m used to reading and writing in English every day because of my job activities. However, I’m not used to practicing English frequently… It’s a cultural problem in my country of origin*.”

These results are in line with the mainstream literature about the impact of language and language diversity on social interaction, “*language not only reflects social context, but it may also influence social interactions within teams*” (Chen et al., 2006, p. 688). Notably, scholars recognize language as the most basic tool of communication between humans (Ahmad, 2018) used to disseminate knowledge throughout the history of humankind. The language supports knowledge sharing through both written material—such as documents—and sense-making (Renzl, 2007). From a knowledge perspective, communication and knowledge exchange require interacting to share experiences, mental models, and technical skills (Nonaka and Takeuchi, 1995). This kind of “socialization” enhances tacit knowledge, the last one passing on between people and not between impersonal media (Argote and Ingram, 2000). Notably, tacit knowledge includes insights, intuitions, and hunches that are difficult to express and formalize. However, it represents the most significant knowledge base in any organization (Buckman, 1998), thus impelling both firms and institutions to establish a communicative environment and promoting collaboration and coordination among employees and work teams (Marks et al., 2000). Establishing personal connections among people is crucial for the tacit KS (Abrams et al., 2003). Not job-related activities help people to feel comfortable since each person relates to each other on more than an instrumental basis. Since each person has some level of concern for the other, trust-building relationship and commitment are likely to emerge, with positive effects on the exchange of experiences among the parties.

Depending on the above, language diversity is expected to mine the share of tacit knowledge among the team members since a low English proficiency makes it difficult and uncomfortable for people to join non-job-related activities.

As some interviewed reported:

“*It was difficult for me to have a personal meeting with partners in Krakow, where we spoke English.”*

*“I’m ashamed of my English because it’s low… I manage to present materials better.”*

When a low English proficiency emerges within the university research team, people reduce their social interactions, limiting tacit knowledge sharing. In such circumstances, learning opportunities from the project and the other members reduce as consequences, negatively affecting the team’s ability to grasp all the available learning chances.

### Language Variations in KS

Dealing with non-native language usually suggests the speakers adopt some kinds of adjustments to make their language and discourse understandable from the audiences (Ahmad, 2018). Thus, in this research, language variations were interpreted as a tool to share knowledge in the best possible way. As mentioned, the TED project involves seven university research teams from five different countries; each team varies from two (Austrian team) to more than five members (Italian team). More than one member from the same nationality is used to attending the meetings, especially when they are organized as virtual meetings. Not surprisingly, *the interviewees refer to code-switching* from one language to another to overcome communication difficulties when they arise. *Code-switching* is commonly recognized as language mixing; people switch from one language to another in the same discourse to convey linguistic and social information (Carter and Nunan, 2001; Ritchie and Bhatia, 2013). The lack of ability to find the right word or transfer in a sentence what the speaker has in his mind triggers the switch to another language, namely the mother tongue language.

Particularly,

“*When I’m trying to say something in English, and I want to tell you what I’m thinking about, It is really helpful to have someone who is really, really good in the language and you can always ask him. In the beginning, after every meeting, I called my colleague and checked with him if what I understood was correct…*”

“*When we have the opportunity to meet each other, and you forgot some words, you can ask people from your country—how is this in English? I forgot about this—And you cannot behave in the same way when you are online, because it is not polite and it’s not probably possible to do things like this.*”

Therefore, switching away from the official team language allows members to overcome communication difficulties and better explain what they want to say. Both interviews and the direct observations show that code-switching is employed within the team to support the knowledge-sharing process instead of limiting it. This result contrasts with what Ahmad and Widén (2018) observed in multinational organizations. Notably, the authors expected that code-switching away from corporate language negatively affects the organizational KS potential between linguistically diverse employees in a multilingual organization because code-switching limits communication with non-native speakers. A very different situation arises within the empirical evidence examined in this paper. TED team members aim to share their own knowledge to get the project aim. Thus, code-switching in project meetings is used to explain better what people have in mind instead of limiting communication. The presence of homogeneous language clusters (clusters of people speaking the same native language) supports this process effectively, code-switching acting as a knowledge bridge between diverse linguistic clusters.

### The Role of Technology

Studies suggest that, from a KS perspective, managing communication and virtual meetings is more challenging than in face-to-face communication (Maznevski and Chudoba, 2000; Maznevski and DiStefano, 2000). In line with the mentioned, interviewees were asked to report any differences between online and face-to-face discussions to achieve the project aims. Notably, we asked them which kind of meeting (virtual vs. face-to-face) makes KS easier and solves problems. In almost all the cases, the respondents recognize that face-to-face meetings support social interactions and debates. Moreover, technical problems like the low quality of Internet connection and voice tone change are recognized to mine the receivers’ capacity to understand the partners’ are speaking of.

Despite this, our results show that both language proficiency and the twofold role of the transfer and receiver of knowledge should be considered when dealing with non-native speakers’ communication. Particularly, people used to speak English more frequently and showing a high language ability report the weaknesses of virtual meetings in terms of KS, virtual meetings limiting discussion and reducing the chances for the speaker to perceive feedback from the audience. Even when they act as receivers, people displaying high language proficiency support the idea that communication technologies reduce the effectiveness of KS, especially the tacit KS, technologies elude non-verbal communication—mainly people’s face and body’s movement.

As some respondents reported:

“*Face-to-face communications are easier than online communication, because during online meetings, sometimes the screen of the participants is very minimal, so I cannot “read” from their face or their expression if they understand* me.”

“*Face-to-face communications are more informal than the virtual ones. In online communication, only one person speaks, and all the others listen to him…when you meet partners in a real room, more than one people participate in the same discussion, debating their perspectives… I feel much more comfortable when we are all together and can discuss our ideas and opinions.”*

By contrast, people declaring having less language ability prefer virtual meetings since online communication “*makes the use of foreign language* easier.” When acting as speakers (ex.: presenting research results or scheduling activities), virtual meetings and presentations allow members to feel much more comfortable:

“*Using online communication is easier for people not so fluently in a foreign language…you feel more protected when you are behind a screen … despite this, I’m aware that meeting the others is crucial for the growth of my personal knowledge*.”

Similarly, the availability of instant voice translators also helps to balance the language proficiency weaknesses, enabling the receivers (team members) to easier understand the partners during the online meetings.

In line with the above, virtual settings appear to be more effective for sharing certain types of knowledge and overcoming certain language barriers. Notably, using a virtual setting supports discussions about the project tasks, scheduling and coordination mechanisms, and the presentations of each member’s activities and output. Similarly, virtual settings enable members not so fluently in English to easier receive and transfer information. However, all the team members (both fluently and non-fluently English speakers) agree that face-to-face and physical meetings are necessary to improve interactions, informal discussions, and more intense KS. Sharing tacit knowledge requires people to interact.

“*When you meet people, I mean when you meet them physically, the communication is much easier and faster and it more informal. At the beginning of the project, of course, we had to knowledge one to each other…in my opinion, it’s important to know people in order to understand what they have in mind, to exchange ideas, and produce something new*.”

## Discussion

In response to our research aim, collected information and direct observations allowed us to develop some propositions on how the use of a common, non-native language influences the KS among the partners belonging to the TED project and how they face the emerging challenges.

Notably, team members, mainly those showing high English proficiency, do not perceive strong difficulties in KS caused by the use of non-native language when dealing with project-related activities. They confirm their opinion even when different accents require more time-spending communication efforts. By contrast, major challenges were reported regarding social relationships; members feel much more comfortable discussing job-related issues than non-job-related topics. Since the team members use English for job purposes, twofold language proficiency may arise, depending on the emerging knowledge domain (job-related vs. non-job-related knowledge domain). In such circumstances, team members do not perceive relevant difficulties in discussing the project tasks. However, they recognize that language ability affects personal and social relationships, requiring more effort when sharing knowledge within non-job-related domains.

**FIGURE 3:**
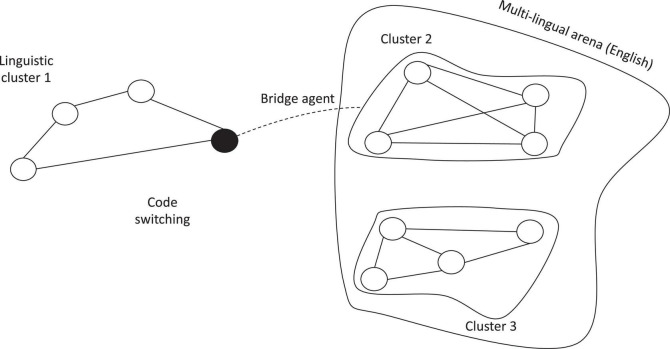
Code switching and bridge agents. Source: Authors’ elaboration.

Moreover, team members perceive cultural diversities to be a more relevant challenge than the lack of lexical and syntactical proficiency, culture shaping people’s behavior and the way—formal or informal—they communicate and relate with the others and the meaning they attribute to concepts and constructs. In the job-related knowledge domain, *divergent interpretations* or *agreement illusions* (perception of consensus when no consensus was achieved—Tenzer et al., 2021) sometimes raised, requiring additional and time-spending efforts to get a mutual understanding of the project goals and how to pursue them. Interestingly, *code-switching* acts as a facilitator of communication and KS within the team. As already mentioned TED project involves seven university research teams from five different countries, each team varying from two (Austrian team) to more than five members (Italian team), language clusters established in a very automatic way. Within TED, language clusters support individuals in their communication process, and *code-switching* allows people to reduce their language weaknesses. Our results contrast with the available literature about the topic. Ahmad and Widén (2015) mainly sustained that language clustering is usually a barrier to KS because it promotes segregation. Moreover, more recently, they stated that *code-switching* away from the corporate language in multilingual situations has negative effects on the knowledge-sharing potential between linguistically diverse employees in a multilingual organization (Ahmad and Widén, 2018). Unlike the authors, we focused on an international university research team aiming to share knowledge to get the project’s final aim. Under some premises, the team members make everything they can to manage linguistic difficulties and overcome the actual barriers to KS. As a result, speakers employing *code-switching* act as a bridge-agent between their linguistic cluster and the English one, supporting common understanding and knowledge diffusion (Figure 3).

More *hidden barriers* established within the non-job-related knowledge domain, respondents often judge the partners’ behavior as “formal,” “informal,” “silent,” or “direct.” Both direct observations and interviews confirm the emergence of cultural-embedded obstacles to KS within the project, even if they were established out from the team members’ awareness. This result finds support in Maznevski and DiStefano (2000), who stressed that cultural values and norms are usually implicit and taken for granted. Their most profound effects on behavior and interaction are usually hidden and extremely difficult to identify and address.

In line with the mentioned considerations, we support the idea that language diversity differently affects KS within the TED project, the effectiveness of KS depending on the language proficiency developed within a specific knowledge domain. Notably, job-related knowledge is likely to be easily shared among the members. When some difficulties arise, mainly in terms of *divergent interpretations* or *agreement illusion*, they depend more on perceived cultural diversity than on the partners’ linguistic ability.

By contrast, in the not job-related knowledge domain, the partners reported more language weaknesses, the last ones depending on both the lack of proficiency in the domain and partners’ cultural background, which reduce personal relationships. From a knowledge perspective, the emergence of cultural-embedded/hidden barriers negatively affects the sharing of tacit knowledge since tacit knowledge transmits exclusively through socialization and social interactions (Lee and Choi, 2003). Because of the mentioned, a low level of tacit KS is expected within the project, severely mining the team capacity to acquire and develop new and innovative outcomes. Based on our results, we detail the following propositions:

Proposition 1: In international university research teams (non-native English members), twofold language proficiency may arise depending on the knowledge domain, namely, job-related vs. non-job-related knowledge domain.
Proposition 2: In the job-related domain, multiple cultural mindsets and different communication behaviors sometimes cause *divergent interpretation* and *agreement illusion* requiring time-spending efforts to share meanings and get the project goals.
Proposition 3: *Code-switching* away from the project language is likely to have positive effects on communication and knowledge flows, speakers acting as bridge agents between their linguistic cluster and the English one.
Proposition 4: Language proficiency and hidden barriers reduce personal and social relationships, hampering the sharing of tacit knowledge within the team and the project.

**FIGURE 4:**
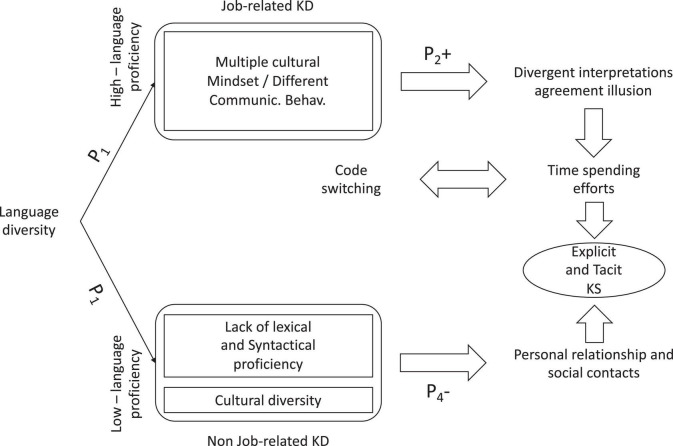
Barriers to KS within TED. Source: Authors’ elaboration.

Summarizes our propositions and depicts our model on the impact of language diversity on KS within the international university research teams (Figure 4).

Because of Pandemic, virtual meetings have replaced physical and face–to–face relationships among the partners. Since early studies have shown how media cause misinterpretation or misunderstanding Klitmøller and Lauring (2013), we examined how team members evaluate technology adoption in terms of communication and KS. Even though almost all the partners agree that physical and direct interactions are preferable to establish more intense and fruitful relationships, a strong connection was detected between virtual teams and language proficiency. Mainly referring to job-related knowledge domain, digital technology, tools, and apps allow people to easily communicate with others, overcoming their linguistic weaknesses. Despite this, some doubts remain about the effectiveness of KS, mainly referring to the more equivocal and tacit components of knowledge.

This perspective allowed us to detect which kinds of challenges people who are used to and aim to produce new knowledge face because of their language ability. Detecting these challenges is necessary to manage them effectively, avoiding loss of relevant knowledge and opportunities for innovation.

## Conclusion and Limitations

For a long time, the impact of language in multinational and multicultural organizations has been neglected since it was considered a component of cultural distance (Barner-Rasmussen and Aarnio, 2011). The scholars’ interest in the issue has been increased in recent years because of globalization and the widespread development of international research teams (Klitmøller and Lauring, 2013). Thus, a wide range of contributions has emerged in dealing with the use of non-native language in multinational organizations (Henderson, 2005; Ahmad, 2018; Ahmad and Widén, 2018; Tenzer et al., 2021). Despite this, very little is known about how language, mainly non-native language, affects KS within international research teams, the last defined as work teams aiming to produce new knowledge. To fill this gap, this exploratory study aimed to explore the impact of language diversity on KS within international university research teams, focusing on the evidence of the TED project. Based on rich qualitative interview data and direct observations, we have shown that language proficiency differently affects KS within the international research project, mainly referring to the nature of the knowledge domains (job-related and non-job-related) and the cultural diversities. We further reported how language proficiency and embedded cultural mindsets reduce people’s propensity to engage in personal and more intense social relationships, thus negatively affecting the sharing of tacit knowledge. As a result, the low innovative performance of the project is expected in the long term.

As with any research study, ours has more than one limitation.

First, the study is based on a sample only targeting academics. They are valid representatives of multinational and multilingual research teams, but they are all working in the university sector; thus, we do not add any information about the impact of language diversity on KS within international research team belonging, for example, to different industries.

Second, our qualitative interviews are supported and integrated by the information collected through direct observation (which is a strength of our investigation), but the project is still running. Thus, we do not know much about the longitudinal aspects of the detected challenges to KS. Do these challenges reduce with partners continuously relating to managing new tasks?

Third, we focused on evident and hidden barriers among non-native speakers. However, a significant role played by culture, and cultural diversity on KS emerged by the interviews, requiring wide investigations relating the cultural dimensions to language diversity and the people’s propensity to relate with others.

Fourth, the Pandemic has strongly affected people relationships, reducing the chances for the members to organize meetings and share their ideas physically. We do not know what will happen when COVID-Pandemic ends.

For the reasons above, future research, improving observations, and detecting changes during the project’s development are strongly recommended.

### Theoretical, Practical, and Managerial Implications

Remarkably, our research results provide for theoretical, practical, and managerial implications.

Since research about language and KS has attracted the attention of the scholars in recent years (Welch and Welch, 2008; Schomaker and Zaheer, 2014; Ahmad and Widén, 2015), this study attempts to deepen the analysis of language and knowledge sharing by focusing on the practice of a non-native language, namely English, for KS. Therefore, from a theoretical perspective, our research results suggest new insights in diversity research, cultural neuroscience, cultural psychology, and knowledge management, detailing how language diversity may support adequate knowledge sharing in international research teams. Moreover, based on a case study analysis, this paper responds to the scarcity of empirical evidence and direct observations due to the organizations’ sensitivity about their privacy. As previously reported, the mainstream mainly deals with the impact of language diversity on KS within for-profit and multinational organizations. By contrast, TED project enabled us to focus on KS in a specific academy context that strongly differs from firms, widening our knowledge about the challenges that non-native speakers face in international university research teams and how they managed them. Our manuscript differs from the available contributions and offers new and original insights.

Moreover, this study will carry significant practical implications regarding how language-based impediments to knowledge sharing within international research teams may be mitigated. It also offers new suggestions to improve international collaborations, in line with the goals pursued by the EU under the Erasmus+ programme—Key Action 2, promoting international cooperation for innovation and the exchange of experiences and know-how. It will help devise and manage knowledge management initiatives for the effectiveness of the European Strategic Partnership financed in education, training, and youth. Our research results clearly show that designing teams with more than one person who shares the same language supports code-switching, code-switching acting as a “bridge” among diverse linguistic clusters. Under such circumstances, code-switching may favor communication and KS among the multiple clusters belonging to an international research team. Intensifying the opportunities of informal relationships may also help improve the partners’ awareness of the existing cultural diversities, detecting the most effective practices to share tacit knowledge.

## Data Availability Statement

The raw data supporting the conclusions of this article will be made available by the authors, without undue reservation.

## Ethics Statement

Ethical review and approval was not required for the study on human participants in accordance with the local legislation and institutional requirements. Written informed consent from the [patients/participants OR patients/participants legal guardian/next of kin] was not required to participate in this study in accordance with the national legislation and the institutional requirements.

## Author Contributions

RC was responsible for sections Materials and Methods and Findings. PM was responsible for sections Literature Review and Discussions. YL was responsible for sections Introduction and Conclusion and Limitations. All authors contributed to the article and approved the submitted version.

## Conflict of Interest

The authors declare that the research was conducted in the absence of any commercial or financial relationships that could be construed as a potential conflict of interest.

## Publisher’s Note

All claims expressed in this article are solely those of the authors and do not necessarily represent those of their affiliated organizations, or those of the publisher, the editors and the reviewers. Any product that may be evaluated in this article, or claim that may be made by its manufacturer, is not guaranteed or endorsed by the publisher.
