# Supplementary material for: The Impact of Language Diversity on Knowledge Sharing Within International University Research Teams: Evidence From TED Project
Source: Front Psychol. 2022 Apr 21;13:879154. doi: 10.3389/fpsyg.2022.879154 (PMC9069179; doi:10.3389/fpsyg.2022.879154)
Supplement: Supplementary file 1 [file Data_Sheet_1.pdf]

APPENDIX 1 – LANGUAGE DIVERSITY AND KNWOLEDGE SHARING

(INTERWIEV GUIDE)

(Source: Ahmad 2018. Questions in red were added in accordance with literature review about the topic)

A) Background of the interviewee

1) Name
2) University
3) Role
4) Length of tenure at your university
5) Native language
6) Other language spoken
7) In which language do you engage in your daily job activities? How frequently do you use English in your job activities?
8) Did you spend some living or working time abroad? If yes, how long did you stay?

B) Participation in International Research Team

1) Are you part of any formal or informal international research team? (With the exclusion of TED)
2) What kind of international projects are you working on? (With the exclusion of TED)
3) Who are the other team members and where are they from? Do you know what their native language is?

C) Language diversity and networking

1) Referring to TED project, what kind of problems do you face and who do you go for advices and suggestions?
2) Does anyone else contact you for advice and information? Describe some specific situations
3) When communicating with other partners, have you experienced challenges due to differences in language and culture? Does your language (or language proficiency) orient your social interactions? Can you describe significant experiences within the TED project?

D) Role of language in knowledge sharing

1) Thinking about TED project, how important is the language (being used) in knowledge-sharing situations or problem-solving discussions?
2) Has there been any incident where the use of English has caused any problems in knowledge-sharing or in team discussions (examples)? Would that have been avoided if it was in your native language?
3) How do you know that the use of English caused issues?
4) What is the difference between using native and non-native languages for information and knowledge sharing?

E) Language variations in knowledge sharing

1) How do you try to deal with these problems caused by language in the above-mentioned knowledge-sharing situations?
2) What kinds of strategies are most effective when sharing knowledge in English language? (Ex. Code switching, convergence, etc.)
3) Do you make any kind of variations in your knowledge-sharing discussion (examples from previously mentioned scenarios) due to the use of English language?
4) When you use English, in your view, what things should be considered for successful exchange of information with your colleagues or in a project meeting?
5) Is your personal or professional experience of working abroad helpful in anyway?

F) Narrative

1) Think about TED meetings that involved sharing of know-how, skills, information. Please describe one such event that according to your view was unsuccessful or did not go very well because of the use of English. Then also describe a successful one.

G) The moderating role of technology

1) Do you detect any differences between *on-line* and *face-to-face* discussions? Considering the adoption of non-native language to reach the project' aims, which kind of meeting (virtual vs face-to-face) does enable you to easier share knowledge or solve problems?
2) When do you experience the same language challenges (mentioned above) when meeting on line?
